# A Multilocus Sequence Typing System (MLST) reveals a high level of diversity and a genetic component to *Entamoeba histolytica* virulence

**DOI:** 10.1186/1471-2180-12-151

**Published:** 2012-07-27

**Authors:** Carol A Gilchrist, Ibne Karim M Ali, Mamun Kabir, Faisal Alam, Sana Scherbakova, Eric Ferlanti, Gareth D Weedall, Neil Hall, Rashidul Haque, William A Petri, Elisabet Caler

**Affiliations:** 1 Departments of Medicine, School of Medicine, University of Virginia, Charlottesville, VA, USA; 2 International Centre for Diarrhoeal Diseases Research, Dhaka, Bangladesh; 3 Rajshahi Medical College, Rajshahi, Bangladesh; 4 J. Craig Venter Institute, Rockville, MD, USA; 5 Institute of Integrative Biology, University of Liverpool, Liverpool, UK

## Abstract

**Background:**

The outcome of an *Entamoeba histolytica* infection is variable and can result in either asymptomatic carriage, immediate or latent disease (diarrhea/dysentery/amebic liver abscess). An *E. histolytica* multilocus genotyping system based on tRNA gene-linked arrays has shown that genetic differences exist among parasites isolated from patients with different symptoms however, the tRNA gene-linked arrays cannot be located in the current assembly of the *E. histolytica* Reference genome (strain HM-1:IMSS) and are highly variable.

**Results:**

To probe the population structure of *E. histolytica* and identify genetic markers associated with clinical outcome we identified in *E. histolytica* positive samples selected single nucleotide polymorphisms (SNPs) by multiplexed massive parallel sequencing. Profile SNPs were selected which, compared to the reference strain HM-1:IMSS sequence, changed an encoded amino acid at the SNP position, and were present in independent *E. histolytica* isolates from different geographical origins. The samples used in this study contained DNA isolated from either xenic strains of *E. histolytica* trophozoites established in culture or *E. histolytica* positive clinical specimens (stool and amebic liver abscess aspirates). A record of the SNPs present at 16 loci out of the original 21 candidate targets was obtained for 63 of the initial 84 samples (63% of asymptomatically colonized stool samples, 80% of diarrheal stool, 73% of xenic cultures and 84% of amebic liver aspirates). The sequences in all the 63 samples both passed sequence quality control metrics and also had the required greater than 8X sequence coverage for all 16 SNPs in order to confidently identify variants.

**Conclusions:**

Our work is in agreement with previous findings of extensive diversity among *E. histolytica* isolates from the same geographic origin. In phylogenetic trees, only four of the 63 samples were able to group in two sets of two with greater than 50% confidence. Two SNPs in the cylicin-2 gene (EHI_080100/XM_001914351) were associated with disease (asymptomatic/diarrhea p = 0.0162 or dysentery/amebic liver abscess p = 0.0003). This study demonstrated that there are genetic differences between virulent and avirulent *E. histolytica* strains and that this approach has the potential to define genetic changes that influence infection outcomes.

## Background

The eukaryotic parasite *Entamoeba histolytica,* the causative agent of amebiasis, is a major cause of morbidity and mortality worldwide, as well as a category B priority biodefense pathogen [1]. In Dhaka, Bangladesh, surveys done in a cohort of children living in an urban slum showed evidence of *E. histolytica* infection (determined by detection of parasite antigen in either diarrhea or monthly surveillance stool) in 80% of the children tested [2]. Host genetics can influence susceptibility to infectious disease and a single amino acid substitution in the host cytokine receptor homology domain 1 of *LEPR* and a difference in the leukocyte antigen class II allele expressed are associated with increased susceptibility to intestinal infection by the *E. histolytica *[3,4]. Symptomatic disease occurs in only a minority of *E. histolytica* infections (20%) in an unpredictable manner and an initially asymptomatic infection can over time convert to invasive disease (~12.5%), amebic liver abscess can occur years after travel to an endemic area [5,6]. It is hypothesized that both host and parasite factors contribute to the outcome of an *E. histolytica*[7]. However, although progress has been made in both the identification and characterization of parasite virulence factors and in understanding the regulation of their gene expression, direct manipulation of the *E. histolytica* genome remains elusive, and the traits affecting parasite virulence have not been genetically mapped [8-17].

Despite this variations that occur within repeat-containing genes in the amoeba genome *chitinase* and serine-rich *E. histolytica* protein *SREHP* have been used to examine the link between *E. histolytica* genetics and disease [18-22]. The high rates of polymorphism however at the loci make it difficult to use them for this purpose and an association between some of these markers and virulence has not been proven in large scale studies [18,21]. However, based on the composition of highly repetitive tRNA arrays, *E. histolytica* has been shown to have distinct genotypes with different potentials to cause disease [23-27].

*E. histolytica* tRNA genes are unusually organized in 25 arrays containing up to 5 tRNA genes in each array, with intergenic regions between tRNA genes containing short tandem repeats (STRs) [27]. A 6-locus (D-A, S-Q, R-R, A-L, S^TGA^-D, and N-K) tRNA gene-linked genotyping system has shown that the number of STRs at these loci differ in parasite populations isolated from three clinical groups (asymptomatic, diarrhea/dysentery and liver abscess) [24,26]. The variations occurring in tRNA genotypes, even between the ameba strains isolated from the intestine and in the liver abscess of the same patient, suggest that not all strains of *E. histolytica* have the same capacity to reach the liver of the infected host [28]. However, the diversity of tRNA linked STR genotypes occurring even in a restricted geographic region, and the frequent occurrence of novel genotypes, limit their usefulness to predict infection outcome or to probe the population structure of *E. histolytica *[25,29,30]. The extensive genetic polymorphism in the repeat sequences of SREHP, chitinase and tRNA arrays for instance could reflect slippage occurring during *E. histolytica* DNA replication as Tibayrenc *et al.* hypothesize that the parasites exist as clonal populations that are stable over large geographical areas and long periods of time [31,32].

Compared with other DNA markers, single nucleotide polymorphisms (SNPs) are genetically stable, amenable to future automated methods of detection, and in contrast to the highly repetitive tRNA arrays, their location can be mapped in the *E. histolytica* genome [33-35]. After the first sequencing and assembly of *Entamoeba histolytica* HM-1:IMSS genome was published by Loftus *et al.* Bhattacharya *et al.* amplified and sequenced 9 kb of coding and non-coding DNA to evaluate the variability of *E. histolytica* SNPs in 14 strains and identified a link between some genotypes and clinical outcome [36]. The advent of the next generation of high throughput genomic sequencing (NGS) technologies has provided more comprehensive opportunities to investigate variation in the genome of *E. histolytica* and clinical outcome by allowing the fast and efficient way to sequence laboratory-cultured ameba of clinical relevance [35,37]. These cultured strains were isolated from different geographical areas endemic for amebiasis and contained large numbers of “strain-specific” SNPs in addition to SNPs present in more than one strain [35]. The sequence variations associated with virulence strains previously identified in the sequenced 9 kb DNA (a synonomous SNP in XM_001913658.1the heavy subunit of the Gal/GalNAc lectin gene (894^A/G^), and SNPs in the non-coding DNA either between XM_652295.1 and XM_652296.2 sequences (236^T/G^, 240^A/G^ and 561^T/G^) or 5’ of the Amoebapore C transcript XM_650937.2 (407^A/C^ and 422^A^) seemed to be present only the two to four Bangladesh isolates sequenced by Bhattacharya *et al.* and were not present in the available international sequenced whole genomes [36].

The goal of this work was to develop a set of less variable markers to profile a large number of strains from different regions of the globe, therefore we selected additional non-synonomous SNPs which Bhattacharya *et al.* had shown to be less variable, to probe the population structure of *E. histolytica* in depth [36]. The new SNPs were present with a frequency of 0.3-0.6 in the pool of geographically disparate *E. histolytica* parasites whose genomes had been sequenced. We restricted our SNP candidates for initial analysis to genes with the potential to be involved in the virulence of this parasite [8-17]. As our current hypothesis is that the development of disease is multifactorial, or polygenic, and involves a combination of parasite factors in the current work we selected several loci to test for their association with disease outcome in *E. histolytica*. These loci contained SNPs that resulted in non-synonomous changes to the encoded amino acids, were present in more than three of the sequenced *E. histolytica* genomes, and enriched either in strains originating from symptomatic or asymptomatic infections. We have shown that two of these SNPs were significantly associated with disease severity in Bangladesh isolates.

## Results

### Initial identification and validation of single nucleotide polymorphisms identified using Next Generation Sequencing

The genome sequencing projects of multiple *E. histolytica* strains performed at the J. Craig Venter Institute (JCVI) and at the Institute of Integrative Biology (University of Liverpool) provided the sequence data used for the identification of SNPs (Table 1) [35]. A total of 10,855 SNPs within coding DNA were identified in the sequenced genomes (Additional file 1: Table S1). Each strain had approximately 1,500 homozygous and 1,000 heterozygous SNPs. Half of all the SNPs identified were unique and present in only one strain (“private” SNPs). Like Ghosh *et al.* we identified mainly dimorphic SNPs, while potential tri- and tetrazygote variants were very infrequent [22]. This, however, may reflect a bias in SNP detection programs because Mukherjee *et al.* observed considerable heterogeneity in the ploidy of *E. histolytica *[38].

**Table 1:** **Genomes sequenced by the Genomic Sequencing Center for Infectious Diseases (GSCID) and the Institute of Integrative Biology,*E. histolytica*Genome sequencing projects**

**Strain id**	**Genbank identifier if available**	**Source/reference**
GSCID *E. histolytica* Genome Sequencing Project		
MS96-3382	885314	R. Haque, unpublished data ICDDR,B
DS4-868	885310	Ali *et al.* 2007 [24]
KU 27	885311	Escueta-de Cadiz *et al.* 2010 [29]
KU 50	885313	Escueta-de Cadiz *et al.* 2010 [29]
KU 48	885312	Escueta-de Cadiz *et al.* 2010 [29]
		University of Liverpool *E. histolytica* Genome Sequencing Project		
HK-9		Ungar *et al.*, 1985 [39]	
PVBM08B		University of Liverpool genome resequencing project [35]	
PVBM08F		University of Liverpool genome resequencing project [35]	
2592100		R. Haque, unpublished data ICDDR,B	
Rahman		Diamond, and Clark. 1993 [40]	
MS84-1373		R. Haque, unpublished data ICDDR,B [35]	
MS27-5030		R. Haque, unpublished data ICDDR,B [35]	

To validate the use of SNPs from next generation sequencing data, a set of 12 SNPs predicted by NGS were verified by conventional Sanger sequencing of PCR amplicons from three selected strains, MS96-3382 (MS indicates monthly stool; this strain was established from an asymptomatic infection), DS4-868 (DS indicates diarrheal/dysenteric stool; this strain was isolated from a symptomatic infection) (sequenced as described in Additional file 1: Table S1) and the reference sequence HM-1:IMSS (Table 2). Primers were designed to amplify the region containing each SNP. The primers used are detailed in Additional file 1: Table S2 and the amplicons are shown in Additional file 1: Table S3 (primer sequences underlined). PCR was performed with these primers on MS96-3382, DS4-868, and HM-1:IMSS genomic DNA as described in materials and methods. The amplified products were separated on a 2% agarose gel and DNA fragments of the correct size were gel purified and sequenced by Sanger sequencing. In all cases the results of the Sanger sequencing of the MS96-3382 and DS4-868 amplicons matched the sequence produced by the NGS (Table 2, Additional file 1: Table S1). The Sanger data from HM-1:IMSS also matched the reference genome however a SNP in the alcohol dehydrogenase gene (gene ID EHI_166490/XM_647170.2) was heterozygous in this HM-1: IMSS reference strain, which was not previously known (Table 2). We therefore concluded that *E. histolytica* single nucleotide polymorphisms studied here were accurately identified.

**Table 2:** **Verification, by Sanger sequencing, of 12 polymorphic loci identified by Next Generation Sequencing (NGS) of *E. histolytica*genomes**

**Strain**		**Reference sequence**	**HM-1:1MSS**		**DS4-868**		**MS96-3382**	
**Genbank accession number**	**Gene id**		**NGS**	**Sanger**	**NGS**	**Sanger**	**NGS**	**Sanger**
XM_644365	EHI_103540	63883^C^	C	C	C	C	C/A	C/A
XM_645788	EHI_069570	120673^G^	G	G	A	A	A	A
XM_647032	EHI_134740	54882^G^	G	G	G	G	A	A
XM_651435	EHI_041950	9878^A^	A	A	A	A	C	C
XM_647310	EHI_065250	10296^C^ 10297^T^	CT	CT	TC	TC	TC	TC
XM_647310	EHI_046600	6048^A^	A	A	C	C	C	C
XM_647170	EHI_166490	28371^G^	G	G/A	G	G	G/A	G/A
XM_652055	EHI_049680	91356^A^	A	A	A	A	C	C
XM_648588	EHI_188130	32841^C^	C	C	T	T	T	T
XM_001914355	EHI_083760	807^T^	T-x-G	T-x-G	T-x-G	T-x-G	T-x-A	T-x-A
		784^G^						
XM_647392	EHI_126120	105607^A^	A	A	A	A	G	G
XM_001913688	EHI_168860	11109^G^	G	G	A	A	A	A

Verification of SNPs identified during Next Generation Sequencing of *E. histolytica* genomes.

### Candidate single nucleotide polymorphisms

The resampling results described above indicated that SNPs were maintained within an *E. histolytica* strain under continuous culture. However, this does not exclude the possibility that a particular genotype may change in frequency within an endemic population. To test for association between SNPs and disease outcome, *E. histolytica* samples were collected from an area endemic for amebiasis (ICDDR and Rajshahi Medical College, Rajshahi, Bangladesh- Additional file 1: Table S4). Both field samples and xenic cultures established from asymptomatic and symptomatic infections were used as a source of DNA (19 amebic liver aspirates; 26 xenic cultures (14 established from asymptomatic infections and 12 from diarrheal); 20 *E. histolytica* positive samples from diarrheal stool; and 19 *E. histolytica* positive samples collected during monthly stool sample surveillance). We anticipated that the virulence of this parasite in humans may not be the direct target of selection, because invasive disease does not seem to confer an advantage to pathogen dissemination [41]. To focus on potentially genetically stable SNPs, which were nevertheless variably present in the different stains, we selected non-synonomous SNPs in the available data that were present in at least four, but not more than nine genomes. This allowed us to select for polymorphic SNPs that frequently occur in ameba and may represent genetically stable or ancestral variants that remain at a frequency of 0.3 to 0.6 a frequency that gave us sufficient statistical power to detect 2x differences within the amebic population surveyed in this study. For a SNP to be considered a candidate for association with symptomatic disease it had to occur at a greater frequency in the isolates from symptomatic amebic infections. Twenty-one potentially informative loci were chosen for further analysis in a larger number of *E. histolytica* isolates as described in the methods section of this paper (Additional file 1: Table S5 and S6).

### SNP genotyping of *E. histolytica* clinical isolates

The 21 marker loci selected from whole genome sequencing data were used to genotype clinical isolates of *E. histolytica*. DNA isolated from three sources, stool samples, short term xenic cultures of parasites from stool and amebic liver abscess aspirates, was used as a template to amplify the 21 loci. PCR products were sequenced using Illumina sequencing technology and the resulting demuliplexed sequence reads aligned to reference sequences representing the genes to which each amplicon corresponds in order to determine the nucleotide(s) present in the sampled genomes (see Additional file 1: Table S7).

Five of the 21 targets were not consistently co-amplified in our PCR reactions. This could have been due to differences in primer efficiency or off-target amplification in the xenic culture and stool specimens that contain an undefined mixture of intestinal microflora or it may also be because the gene is missing from some isolates or highly divergent. These five loci were not included in later analyses that only used the 16 remaining loci. We selected only those samples where all target sequences were efficiently amplified and we observed good coverage of all the expected amplicon sequences. If one or more of the targets was missing, then the sample was eliminated (Additional file 1: Table S7). The final data set consisted of 63 or the original 84 samples (63% of asymptomatically colonized stool samples, 80% of diarrheal stool, 73% of xenic cultures and 84% of amebic liver aspirates) which passed quality control and had the greater than 8 fold sequence coverage needed to confidently call SNPs.

**Figure 1:**
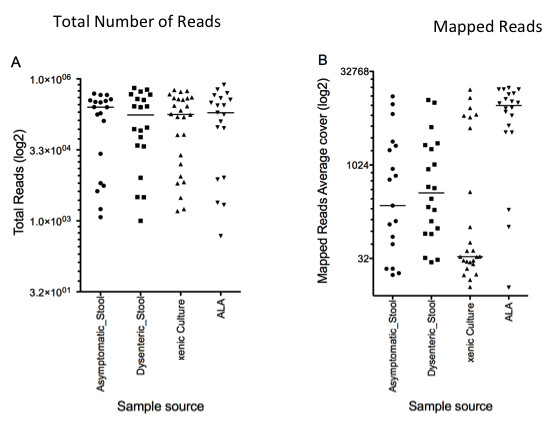
**Amplicon sequencing efficiency for individual samples.A**) Number of reads obtained from the Illumina libraries prepared from different sample source x-axis libraries prepared from different sample source; y-axis number of reads (log2 scale) **B**) Average coverage of the reads when mapped to the concatenated amplicon reference; x-axis libraries prepared from different sample source y-axis average coverage of mapped reads (log2 scale) Line indicates median number of reads.

The libraries generated from stool samples and from polyxenic culture contained a greater number of reads that did not map to the *E. histolytica* amplicons than those obtained from amebic liver abscess aspirates. This was likely due in part to off-target amplification (Figure 1) of gut flora, or a reduction in specificity because most of these samples did not undergo nested PCR amplification prior to library preparation. Samples isolated from amebic liver aspirates do not have associated bacterial flora, unlike pyloric abscesses, therefore a higher proportion of the template DNA is *E. histolytica*.

**Figure 2:**
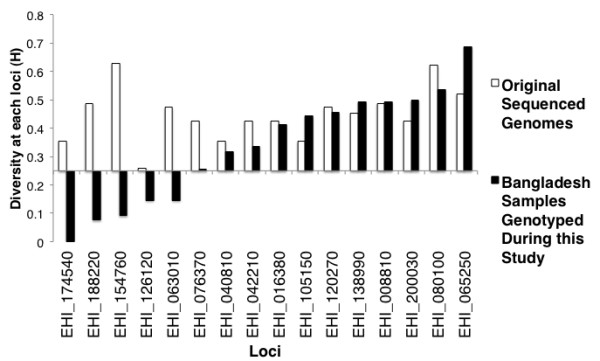
**Similarity of *E. histolytica* diversity in Bangladeshi and whole genome sequenced strains.** Shown on the y axis (H) is the calculated heterozygosity and represents sum of the squared allele frequencies was subtracted from 1 on the x axis the loci containing the SNPs genotyped by MSLT(■ value in Bangladesh samples genotyped during this study, (□ value in the sequenced genomes described in Table 1).

In the samples that passed quality control, the read depth for individual SNPs was >8x coverage; this was considered adequate for SNP verification. SNPs were scored as described in materials and methods. The results of the illumina sequencing and the presence of predicted and novel SNPs within the amplicon sequences was tabulated as homozygous Reference (the same as the reference HM-1:IMSS sequence at this position) heterozygous (contained both the HM-1:IMSS nucleotide and the variant nucleotide at this position) or homozygous Non-Reference (has only the variant base at this location) (Additional file 1: Table S8). In Figure 2 the diversity of the SNPs at each locus in both the original sequence data (genomes shown in Table 1), and in the Bangladesh samples analyzed in this study, (extra details shown in Additional file 1: Table S9).

**Figure 3:**
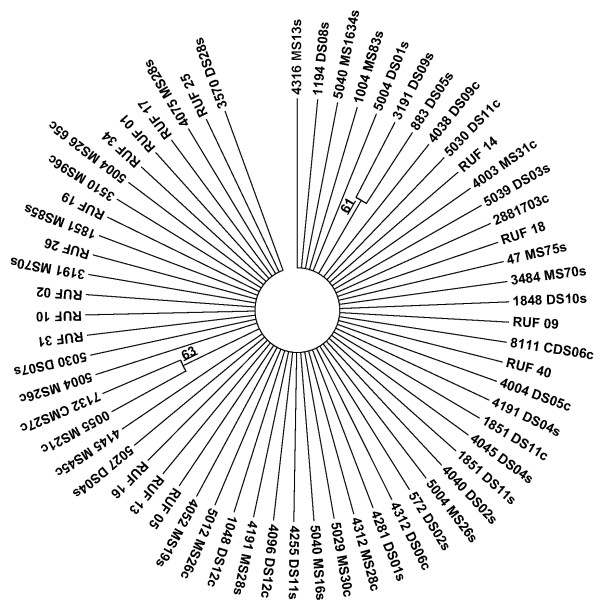
**Lack of consistent patterns of descent among SNP markers from Bangladeshi *E. histolytica*isolates suggests they segregate independently.** Consensus phylogeny inferred from 100 bootstrap replicates of polymorphic SNP markers, constructed using the MEGA 5 program and the Maximum Likelihood method based on the Tamura-Nei model and using the sequences shown in Additional file 1: Table 8 [42]. Branches produced in fewer than 50% of the bootstrap phylogenies were collapsed. Sequences from stool have the suffix s; culture c; monthly survey stools begin with MS or CMS, diarrheal DS or CDS, amebic liver abscess samples RUF.

Our work supports previous finding of extensive diversity among *E. histolytica* isolates from the same geographic origin, in phylogenetic trees only four of the 63 samples could be grouped into two sets of two with greater than 50% confidence suggesting that the genotypes of individual parasites do not contain consistent phylogenetic signals (Figure 3). Such a result suggests that the markers do not share the same genealogy, likely due to extensive recombination or re-assortment breaking down linkage between markers. The diversity of *E. histolytica* genome raises a concern in regard to later analysis as it raises the possibility that a rapid rate of evolution may drive any observed differences between *E. histolytica* genotypes in samples isolated in regions separated even by relatively small geographical distances.

### The effect of adaptation of to *in vitro* culture on SNP allele frequencies

To examine the potential effect of adaption to *in vitro* culture on the frequency of SNP alleles, and therefore how well transiently or long established cultured trophozoites represent the parasite population, SNP allele frequencies were compared between parasites genotyped directly from stool samples and those from cultured trophozoites (Additional file 1: Table S10).

**Figure 4:**
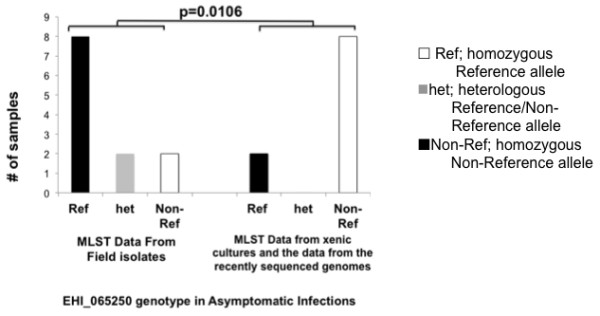
**Amebic culture effect on the EHI_065250 *Entamoeba* genotype.** Distribution of the EHI_065250 SNP at the 10296 location in field isolates or cultured strains established from asymptomatic disease (p = 0.0166). The distribution of the individual SNPs, which were either Reference (Ref), Non-Reference (Non-Ref) or heterologous was shown on the x-axis. The number of samples of with this genotype isolated from patients with asymptomatic disease was shown on the y-axis.

In cultures originating from asymptomatic isolates five linked Non-Reference SNPs at the LCAT EHI_065250/XM_647310.1 locus were detected in 80% of the strains, these same SNPs occurred in only 16% of the *E. histolytica* positive stool samples from asymptomatic hosts (Figure 4). This suggests that during establishment of *E. histolytica* cultures a strong selection pressure was exerted on sequence in linkage with the LCAT EHI_065250 gene. This could either cause growth failure of the strains with the Reference allele or the outgrowth of a minority genotype in mixed infections (previous studies using the short tandem repeats have indicated that mixed infections are rare however this possibility cannot be discounted [24]).

To confirm that this change in the distribution of the Non-Reference SNPs was due to culture conditions, and not due to the Bangladesh origin of these samples, the comparison was repeated using only the strains isolated and transiently established in culture at ICDDR, B and genotyped using MLST during this study. A similar trend was observed.

EHI_065250 (LCAT) belongs to a gene family that consists of ten genes; they range in identity from 82% to 51% (Additional file 2: Figure 1A and B); two are highly similar to EHI_065250 (82 and 81% identity). The primers used in SNP amplification were specific for EHI_065250 and did not amplify the other members of this gene family. The other LCAT gene sequences are sufficiently different that off-target amplification would be detected in the sequence alignments of the Illumina reads. Such off-target amplification was never observed, confirming that amplification was specific for the target EHI_065250 locus.

The effect on SNP genotype was only apparent for the LCAT EHI_065250 SNPs and the p value of the EHI_065250 SNPs was not sufficiently low to eliminate the possibility of false discovery (q value = 0.32, Additional file 1: Table S10). Therefore the cultured strains were included in Table 3 and the statistical association of SNPs with disease phenotype was determined using the complete dataset but confirmed using the data set with only clinical samples (Additional file 1: Table S11 Data Set 1 and 2).

**Table 3:** Association of SNPs with disease phenotype

					**Significance of SNP distribution in Invasive amebic liver abscess, dysentery and Asymptomatic disease**	
**Genbank**^**#**^**accession number**	**AmoebaDB ID**	**Non-synonomous substitution**	**Location in reference contig**	**SNP**	**p value**	**q-value**
XM_647889.1^&^	EHI_080100	Pro361Leu	2725^C/T^	1	0.002**	0.032**
XM_647310.1^&^	EHI_065250	Ser399Asp	10296^A/G^	3	0.05**	0.3
			10297^G/A^	4		
XM_644633.2	EHI_200030	Leu60Ile	16181^C/A^	8	0.08	0.31
XM_646031.2	EHI_120270	Pro21Ser	7994^C/T^	9	0.10	0.31
XM_647889.1	EHI_008810	Leu326Ile	73463^C/A^	10	0.24	0.44
XM_643253.1	EHI_040810	Ala197Glu	1216^C/A^	11	0.31	0.46
XM_645270.1	EHI_105150	Ile282Met	27395^T/G^	12	0.42	0.56
XM_001913781.1	EHI_138990	Val1288Leu	30231^G/T^	13	0.52	0.64
XM_651449.1	EHI_042210	Pro58Leu	39051^C/T^	14	0.92	1.00
XM_648423.2^&^	EHI_016380	Tyr702His	17795^T/C^	15	0.97	1.00

^#^Only loci with diversity H value over 0.25 shown.

** <0.05.

^&^Representative SNP chosen in linked SNP data sets.

### Genetic differences between virulent and avirulent *E. histolytica* strains

**Figure 5:**
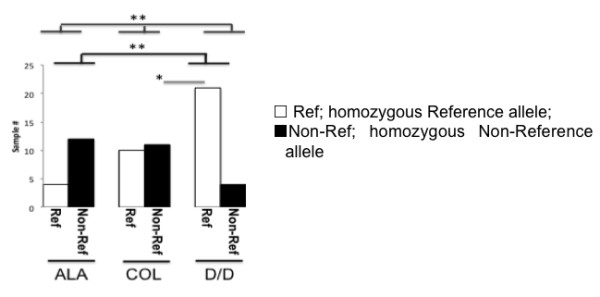
**SNPs 1&2 in the EHI_080100 locus segregate with disease.** Distribution of the SNP1 which was either Reference (□,MS)(Ref), Non-Reference (■ ALA);(Non-Ref) was shown on the x-axis. The number of samples of with this genotype isolated from patients with either amebic liver abscesses diarrhea/(D/D) asymptomatic disease COL was shown on the y-axis. Fisher’s pairwise comparison between asymptomatic and diarrhea/dysentery p = 0.0182 (*); between amebic liver abscess and diarrhea/dysentery samples p = 0.0003; q = 0.0144 (**); Chi-squared contingency analysis of all phenotypes p = 0.002; q = 0.032 (**).

The EHI_080100/XM_001914351.1 cylicin-2 locus contained two closely linked SNPs 1&2. These SNPs were significantly associated phenotype (Non-Reference SNP was present in 75% of ALA samples; positive samples or cultures isolated from the monthly survey stool 52% and in 16% of samples or cultures isolated from diarrheal stool; p = 0.002; q = 0.032; Figure 5). Both the Reference and the Non-Reference variants at this locus were present in asymptomatic samples however the Non-Reference variants were more frequent in liver abscess samples and less frequent in samples isolated from patients with diarrhea/dysentery (Figure 5 pair wise comparison between asymptomatic and diarrhea/dysentery p = 0.0182 and between amebic liver abscess and diarrhea/dysentery samples p = 0.0003; q = 0.0144).

Amebic liver abscess is a complication only found in adults whereas dysentery is more frequent in children. The liver aspirate samples in this study were collected from adults, at Rajshahi Medical College Hospital, Bangladesh. This is a geographically distinct location from the dysenteric and asymptomatic samples that were collected from children in Dhaka, Bangladesh. One goal of this study was to identify SNPs to type the virulence potential of the parasite in amebic liver aspirates; if SNPs occur at different frequencies in Dhaka and Rajshahi isolates they will appear as potential biomarkers of parasites with the potential to initiate amebic liver abscesses. The difference in SNP 1&2 frequency in both asymptomatic and diarrheal samples was replicated however in the sequenced genomes from diverse populations in Asia and South America (described in Table 1 and Additional file 1: Table S6 and included in Data set 2 Additional file 1: Table S11) [24,29,35,39].

The previously discussed locus, LCAT EHI_065250, which contained five different SNPs (3–7), was also associated with symptomatic disease however possible selection in culture rendered the distribution less significant within the larger data set (Table 3).

**Figure 6:**
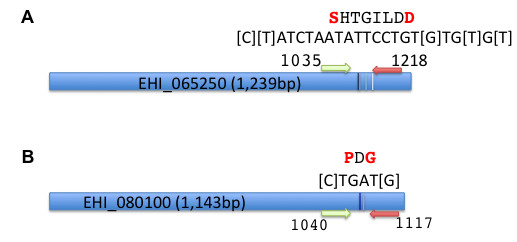
**The locationof the SNPs1&2 in EHI_080100 and EHI_065250 genes.** Mapping of the informative SNPs within the coding sequences. **A**) EHI_065250 and **B**) EHI_080100 genes. Nucleotide position of the amplicon 5’ and 3’ bases are shown and approximate location of the 5’ (green) and 3’ (red) and the positions and number of the targeted SNPs indicated by vertical lines. The bases involved are bracketed in the nucleotide sequence at this region (shown above). The amino acid sequence with changed residues in red is also shown.

The changes at both the LCAT EHI_065250 and the cylicin-2 EHI_080100 loci altered a potential phosphorylation site in the encoded protein sequence (NetPhos [43]), and are located at the C-terminal portion of the proteins (Figure 6). Expression of EHI_065250 has been shown to be modulated in the mouse model of amebiasis, and to be under the control of the URE3-BP transcription factor [9,44]. EHI_080100 appears to be a novel member of the *E. histolytica* “promoter family” potential membrane proteins regulated by the transcription factor URE3-BP with highly similar promoters, and amino- and carboxyl-terminal sequences (sites of signal peptide and transmembrane domains) [44]. EHI_080100 encodes a hydrophilic Glutamic acid/Lysine rich protein with an N-terminal Signal P and although annotated as cylicin-2, it is not an ortholog of the human gene [45]. Its function remains unknown.

## Discussion

*E. histolytica* SNPs were identified in amebic DNA isolated from a Bangladesh population by amplicon sequencing. Non-Reference SNPs in the EHI_080100 cylicin-2 gene were significantly associated with the virulence phenotype (amebic liver abscess > asymptomatic > diarrhea or dysentery).

We initially analyzed the genetic diversity among 12 sequenced *E. histolytica* genomes that represented different geographical origins and disease manifestations, and selected a set of 21 polymorphic sites in coding regions where SNPs change the encoded amino acid. The distribution of these 21 non-synonymous SNPs in field isolates and cultured strains of *E. histolytica* were examined in samples collected from an endemic area in Bangladesh by multilocus sequence typing (MLST). Of 16 loci that passed quality control five were invariantor very infrequent in Bangladesh.

Our results are inconsistent with a model of clonality in *E. histolytica* populations. In a clonal population we would expect to see strong linkage disequilibrium between markers, since linkage would not be eroded by recombination and sexual reassortment. In fact, we saw only two identical genotypes in our sample, suggesting a considerable amount of recombination and/or reassortment. Our results support previous observations, based on short tandem repeat DNA sequences, of high diversity among genotypes even within limited geographical areas [18,21,24]. Due to this complexity, the number of whole genomes sequenced in the pilot studies, were not sufficient to predict accurately the SNPs associated with disease. However, 2 out of the 16 loci examined,(EHI_065250 and EHI_080100), were significantly associated with disease in isolates collected in Rajshahi and Dhaka, Bangladesh.

One caveat to this study was that the amebic liver abscess samples were collected in Rajshahi but the stools samples were collected at a different location (Mirpur, Dhaka); the differences in the grouping of liver abscess and stool *E. histolytica* could reflect geographical differentiation [24]. Ali *et al.* have however previously described different genotypes in liver abscess and enteric samples from the same patients [28]. This suggests a possible genetic selection for parasites with invasive capabilities. Based on our data we suggest a divergent rather than sequential model of the potential to cause severe disease [46]. Non-Reference EHI_080100 SNPs were more frequent in asymptomatic than diarrhea/dysentery samples. However the Non-Reference SNP potentially predisposed the asymptomatic infection to initiate an amebic liver abscess rather than amebic colitis (p = 0.0182) as the Non-Reference EHI_080100 SNPs, were present with even higher prevalence, in samples from amebic liver abscess (p = 0.0003, q = 0.0144). Additional studies are needed to identify additional amebic biomarkers associated with invasive disease.

In both EHI_065250 and EHI_080100 the consequence of the Non-Reference polymorphisms was to change two amino acids within the C-terminal domains. The reason behind the association of these SNPs with invasive disease is not yet clear. The polymorphic genes have not previously been associated with a virulent phenotype, and other than the previously discussed change in at a potential phosphorylation site, there were no other predicted changes in protein function using the currently available bioinformatics tools (PolyPhen http://genetics.bwh.harvard.edu/pph2/http://sift.jcvi.org/www/SIFT_seq_submit2.html) [47,48].

EHI_080100 (cyclin-2) is present on a short region of contiguous DNA in the *E. histolytica *HM-1:IMSS genome assembly that could not be assembled into a larger contiguous DNA segment or sequence scaffold (Table 4). This suggests that the gene may be present in proximity to highly repetitive regions that prevent unambiguous assembly. Lorenzi *et al.* suggest that repeats and repeat-clusters are found at syntenic break points between *E. histolytica* and *E. dispar* and could act as recombination hot spots promoting genome rearrangement [49]. This “informative” locus could therefore reside in regions of DNA prone to allelic imbalance. In addition, no *E. dispar* homologue has been found for EHI_080100, making this gene an interesting candidate for further studies.

**Table 4:** Locations of informative SNPs

**Gene id**	**Contiguous*E. histolytica *DNA region ID**	**Length (bp)**	**Location of SNP(s) (bp)**
EHI_080100	DS571720	5179	2725-2730
EHI_065250	DS571302	38246	10296-10318

Genomic Location of the SNPS in the EHI_080100 and EHI_065250 genes.

The currently identified SNPs could act as genetic “markers” in incomplete linkage disequilibrium with neighboring DNA that contains causative or regulatory SNP (r-SNP) mutations that result in a modulation of gene expression. It is interesting to note that contiguous with the EHI_065250 gene is one of the genes encoding the intermediate subunit of the Galactose- and N-acetyl-D-galactosamine (Gal/GalNAc) inhibitable lectin (igl2) [50]. The Gal/GalNAc inhibitable lectinis a well-characterised virulence factor in *E. histolytica*[51].

It is also possible that amino acids changes resulting from the SNPs directly influence the biological activity of the encoded protein and that these changes affect the ability of the trophozoite to invade its host. What has never been clear is the advantage to the *E. histolytica* parasite to the causation of invasive disease [41]. It is conceivable that these SNPs result in a maladaptation of the coevolving genomes of parasite and host and are the reason for the expression of the virulent *E. histolytica* phenotype [3,4]. The effect of the changes described in this study on the stability and function of the encoded protein is currently under investigation*.*

## Conclusions

*E. histolytica* does not follow the model of *T. gondii* that exists predominantly in a few main lineages [52]. Rather, even in population from a single geographic location, majority of the individual parasites show unique genotypes. The number of tRNA-linked genotypes discovered, are likely to continue increasing in number and will enable the measurement of strain diversity. However, the results presented in this work support the hypothesis that a relationship exists between the genotype of an *E. histolytica* strain and parasite virulence. Unlike the tRNA-linked sequence types (Ali et al, 2012) which are merely surrogate markers for the prediction of infection outcomes, non-synonymous SNPs detected in the present study shows promise to identify parasite factors directly linked to infection outcomes [26]. Although preliminary, our findings identified two candidate genes that may contribute to the pathogenesis of these parasites. The level of genetic variation we observed increases the importance of the SNPs we have linked to disease. We are currently investigating the impact of the non-synonomous changes on the function of these proteins.

To fully understand the genetics of this parasite, additional biomarkers will be needed to understand virulence and different outcomes of the disease at the genome level. In the absence of stable clonal populations deeper characterization of the variation in the *E. histolytica* genome requires sequences from additional ameba strains. Using the protocol described in this paper usable sequence data was gathered from approximately half of the field samples. This allowed the testing of the association of selected candidate SNPs within an endemic population. Given the large amount of variation that occurs, SNPs need to be carefully chosen to type the virulence potential in an *E. histolytica* MLST schema rather than to reflect parasite phylogeny. Future studies are needed which focus on the genome of the infecting parasite in conjunction with the genome of the infected host.

## Methods

### Ethical approval

The Ethical Review Committee at ICCDR,B approved this study. Written informed consent was provided by all study participants and/or their legal guardians.

### Cultured *E. histolytica* strains used for genotyping

*E. histolytica* trophozoites isolated from patients of all age groups seen at the hospital for diarrheal diseases, or from children living in an urban slum area in Dhaka were established in culture at the International Centre for Diarrhoeal Diseases Research, Bangladesh (ICDDR,B). Polyxenic cultures were maintained in biphasic Robinson's medium at 37°C (listed in Additional file 1: Table S4) [53].

### *E. histolytica* DNA derived from stool samples

Recently collected stools from patients of all age groups seen at the hospital for diarrheal diseases, or from children living in an urban slum area in Dhaka were examined macroscopically for the presence of blood and mucus; a smear of feces in 0.9% saline was examined microscopically for the presence of erythrocytes, leukocytes, and *E. histolytica* trophozoites. The DNA was extracted using a slightly modified QIAamp DNA Stool Mini Kit protocol (Qiagen Inc., Valencia, CA) as described previously for specimens from ICDDR,B [54]. Stool samples are also listed in Additional file 1: Table S4.

### *E. histolytica* DNA derived from Amebic Liver Abscess (ALA) aspirates

Aspirates from patients with amebic liver abscesses were obtained only from adults because ALA is an extremely rare complication in children [55]. A presumptive diagnosis of ALA was based on clinical picture, ultrasound examination and positive serology using an *E. histolytica* antigen based ELISA (TechLab *E*. *histolytica* II) [6]. Abscess fluid was obtained under ultrasound guidance from patients with ALA and was purified using the modified QIAamp DNA Stool Mini Kit protocol described above (samples are listed in Additional file 1: Table S4) [6].

### Primer design

Primers for these experiments were designed using the publically available Primer3 program and checked for specificity using the NCBI Primer-BLAST tool [56] (http://www.ncbi.nlm.nih.gov/tools/primer-blast/). All primers used in this study are listed in either Additional file 1: Table S2 or Table S4.

### Whole genome sequencing of axenic cultured *E. histolytica* strains

Whole genome sequencing of five of the *E. histolytica* strains used in this study was carried out at the J. Craig Venter Institute. These sequence traces are deposited  athttp:// http://www.ncbi.nlm.nih.gov/bioproject/9532 dbSNPs Genbank (http://www.ncbi.nlm.nih.gov/projects/SNP/) and AmoebaDB (http://amoebadb.org/amoeba/)[57,58]. This project is also fully described at the NCBI Bio Project page (Accession: PRJNA9532). Whole genome re-sequencing was performed at the Institute of Integrative Biology, (Centre for Genomic Research) University of Liverpool and results deposited at AmoebaDB [35,57]. For a complete list of *E. histolytica* genomes, sequencing technology and Sequencing Center see Table 1 and Additional file 1: Table S1.

### SNP detection and selection of candidate informative SNPs

For genome-wide SNP detection at JCVI the sequenced strains were analyzed using the CLC Genomics Workbench 4.0.2 SNP detection component as described below (see SNP detection and validation of amplicon sequences). In genomes sequenced at the Centre for Genomic Research, SNPs were identified according to the methods described Weedall *et al.* [35]. For a list of the SNP detection method used in each genome see Additional file 1: Table S1. SNPs are listed in Additional file 1: Table S5. The default in the table was Reference and only high confidence SNPs (sequence coverage >8) were identified as Non-Reference. At selected locations a visual inspection of available sequence traces was performed to identify lower confidence SNPs (Additional file 1: Table S6). To identify “ancestral” or genetically stable SNPs we selected SNPs that were present in more than three strains. To pick out SNPs linked to disease the SNPs were grouped according whether the sequenced genome was first isolated from patients with asymptomatic or symptomatic disease. The list of weighted selection criteria included whether the SNPs enriched asymptomatic or symptomatic isolates, if the SNP was present in repeat regions or large *E. histolytica* protein families, whether it was contained in genes with any potential role in virulence, or if orthologous sequences were present in the non-pathogenic but closely related species *E. dispar *[37]. The selected SNPs are shown in Additional file 1: Table S6.

### Preliminary amplicon sequencing and validation

PCR amplifications were performed on a C1000 Thermal Cycler (Bio-Rad) using the High Fidelity Phusion DNA polymerase Master Mix (Finnzymes). Sample DNA (0.5 μl) was added to a 25 μl reaction mix containing 125 pm of the designated primers (5 nM). After an initial denaturation step of 98°C, denaturation at 98°C for 10 sec, annealing of primers at 50°C for 30 sec and elongation at 72°C for 30 sec was performed for 34 cycles. This was followed by a final extension at 72°C for 10 min. The amplified products were separated on a 2% agarose gel and the DNA fragments of the correct size were gel purified and sequenced by Sanger sequencing (GENEWIZ, Inc).

### PCR amplification of SNP markers and preparation ofmuliplexed sequencing libraries

For clinical samples and low copy number culture material, amplicons were generated by nested PCR (see Additional file 1: Table S2 and S3). PCR amplifications were carried out using Phusion High Fidelity DNA polymerase Master Mix (Finnzymes). 1 μl of first round amplified DNA was used as template for the second round of amplification, using the same conditions as for the first round PCR with the exception that the annealing temperature was increased to 60°C and the nested PCR primers were used with tails that contained the unique “barcode” sequences and adaptors necessary for Illumina paired-end sequencing, as described by Meyer and Kircher (Additional file 1: Table S4) [59]. DNA from cultured parasites was used directly as template for the second round PCR amplification only, as its more abundant template made nested PCR unnecessary.

After this step, the different PCR products amplified from original samples were pooled in groups of 5 or 6 and one μl was amplified using 200 nM of the IS4 primer and an indexing primer (Additional file 1: Tables S2 and S4) for an initial denaturation step of 98°C, denaturation at 98°C for 10 sec, annealing of primers at 60°C for 20 sec and elongation at 72°C for 20 sec was performed for 34 cycles. This was followed by a final extension at 72°C for 10 min.

The final amplification product was pooled and purified using the Qiagen MinElute 96 UF PCR purification kit according to manufacturer’s directions, and an aliquot of the amplified library and the accompanying negative controls were run on a 2% agarose gel for quality control. The final library was pooled and DNA concentration determined using a Quant-iT Kit (Invitrogen). Prior to submission for sequencing the size distribution of the DNA in the pooled library sample was examined for insert sizes and confirmed to be of the expected range (200–300 bp) using an Agilent 2100 bioanalyzer.

### Illumina paired-end sequencing of amplicons containing SNP markers

An aliquot of the multiplexed libraries (5 pmol) was denatured and then processed with the Illumina Cluster Generation Station at the J. Craig Venter Institute, Rockville, MD (JCVI, MD, USA), following the manufacturers protocol. Libraries were sequenced on an Illumina GAII,run for 100 cycles to produce reads of 100 bp. Images were collected over 120 tiles (one lane) which contained 715,000 ±60 clusters per tile.

### Data filtering and analysis pipeline

After the run image analysis, base calling and error estimation were performed using Illumina/Solexa Pipeline (version 0.2.2.6). Perl scripts were used to sort and bin all sequences according to indexes CASAVA 1.6 (Illumina).

### Alignment of sequence reads and SNP typing

Amplicon sequence analysis was performed using the high-throughput sequencing module of CLC Genomics Workbench 4.0.2. Raw read output for each indexed amplicon set (derived from samples as indicated in Additional file 1: Table S4) was cleaned by trimming of adaptor sequences, ambiguous nucleotides and low quality sequences with average quality scores less than 20. The remaining reads were used for reference assembly. To assess the level of redundancy and non-specific alignment in each individual dataset, an initial reference-based assembly was executed using the whole *E. histolytica* HM-1:IMSS reference genome (Genbank accession AAFB00000000). As some level of non-specific alignment occurred, the alignment conditions utilized for the final mapping of Illumina reads to the reference assembly were adjusted to require a global alignment of 80% identity over at least 80% of the specific concatenated reference assembly of the target sequences (see Additional file 1: Table S3). Default local alignment settings with mismatch cost of 2, deletion cost of 3 and insertion cost of 3 were used. Reads that were not assembled into contigs in the reference assembly were not analyzed. Consensus sequences derived from the reference assemblies for each amplicon set were utilized for SNP scoring and further phylogenetic analysis.

SNP detection in the amplified DNA was performed using CLC Genomics Workbench 4.0.2 SNP detection component, which is based on the Neighborhood Quality Standard (NQS) algorithm [60]. To identify quality SNPs, putative SNPs were screened following specific criteria based on the read depth, minor allele frequency (10%), the quality of flanking regions and absence of other SNPs within 15-bp flanking regions. SNPs located in repetitive regions were also not considered. The central base quality score of ≥30 and average surrounding base quality score of ≥20 were set to assess the quality of reads at positions for SNP detection. A minimum coverage of 10 and a minimum variant frequency of two was required, and the variations compared against the reference sequence were counted as SNPs. The NQS algorithm looked at each position in the genome alignment to determine if there was a SNP at that position.

### Statistical analysis

The sequences spanning the SNPs were extracted and the IUB base code guide used to describe heterologous bases (see Additional file 1: Table S8). At each locus the sum of the squared allele frequencies was subtracted from 1 to gauge the diversity (heterozygosity) in both the original sequenced genomes and the new MLST data (Figure 2). The *E. dispar* Mercator whole genome alignment deposited in AmoebaDB was used to obtain the equivalent sequences where they existed in this related species (Additional file 1: Table S8) [57,61]. The statistical significance of SNP distribution or genotype group versus the phenotypic manifestation of disease (asymptomatic/diarrhea or dysentery/amebic liver abscess) was determined by use of a Chi-squared contingency test or Fisher’s Exact test using the Prism 5 program (GraphPad Software) and the resulting p values were corrected for multiple comparisons by use of the false discovery rate formula of Benjamini and Hochberg in the R program FDR online calculator made freely available by the SDM project [62,63]. To obtain the correction for multiple comparisons in the pairwise comparison the p-values of all possible combinations (i.e. asymptomatic vrs dysentery; asymptomatic vrs amebic liver abscess; dysentery vrs amebic liver abscess) for a given data set were combined prior to correction. A FDR of 10% was considered significant (http://sdmproject.com/utilities/?show=FDR_).

## Competing interests

The authors have no competing interests to declare.

## Authors’ contributions

CAG conceived, designed, performed experiments, analyzed data and wrote the manuscript. WAP, IKMA, RH, and EC participated in the design of the study and also helped to write the manuscript. IKMA also preformed experiments. MK and FA collected samples and prepared DNA. SS, EF and EC conducted the next generation sequencing of amplicons and analysis of the resulting sequence data. GDW, NH and EC sequenced all genomes and discovered all SNPs described in this study. GDW helped in the writing of the manuscript. All authors read and approved the final manuscript.

## Supplementary Material

Additional file 1 **Supplemental Tables. **This file includes all supplemental tables mentioned in the text in an excel spreadsheet. Click here for file

Additional file 2 **Figure S1. This word document file (.dox) includes Additional file ****2****: Figure S1. describing the LCAT superfamily.** Click here for file
