# Supplementary material for: A Multilocus Sequence Typing System (MLST) reveals a high level of diversity and a genetic component to Entamoeba histolytica virulence
Source: BMC Microbiol. 2012 Jul 27;12:151. doi: 10.1186/1471-2180-12-151 (PMC3438053; doi:10.1186/1471-2180-12-151)
Supplement: Additional file 2 — Figure S1. This word document file (.dox) includes Additional file 2: Figure S1. describing the LCAT superfamily. [file 1471-2180-12-151-S2.docx]

**Supplemental Figure – 1 *E. histolytica* LCAT gene family**

**A**

**
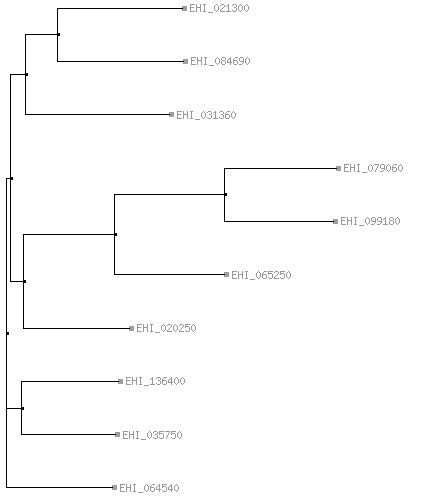
**

**B**

EHI_079060+300_|length=1089rev -------------------------------------------------- EHI_099180reversecomplement TCAATTGCAAACATAAGAATAAACTTCTTCATATACTGCTTTACTATCTA EHI_065250reversecomplement TCAATTGCAAACAAAAGGATAAACTGCTTTATATGATGCTTTACTATCTA

EHI_079060+300_|length=1089rev -------------------------------------------------- EHI_099180reversecomplement ATATTCCCGTGTGTGTATATTTTCCTAAATTTTTAAATGTTGTTGCTCCA EHI_065250reversecomplement ATATTCCTGTGTGTGTATATTTTCCTAAATTCTCAAATGTCTCTGCTCCA

EHI_079060+300_|length=1089rev ------------------------------------------------TC EHI_099180reversecomplement ATATTCTTACAAAATTCTAATGAGTTCAAATTTACTGTTTCATCTCCATC EHI_065250reversecomplement ATATTTTGACAAAATTCTAATGAATTCAAATTAACCGTACCATCTCCATC

**

EHI_079060+300_|length=1089rev ACTATATAAAAGTTTACTGTCACTATCATAACTTTCATTTTCCATTTCTA EHI_099180reversecomplement ACTATATAAAAGTTTACTGTCACTATCATAACTTTCATTTTCCATTTCTA EHI_065250reversecomplement ACTATATAAAAGTTTACTGTCACTATCATAACTTTCATTTTCCATTTCTA

**************************************************

EHI_079060+300_|length=1089rev CACCTTTAATACTTTCATATCCTGATGATATCATACATTGCATTTTAATA EHI_099180reversecomplement CACCTTTAATACTTTCATATCCTGATGATATCATACATTGCATTTTAATA EHI_065250reversecomplement CTCCATCAATGCTTTCATGTCCTGATGATATTAAACAATGCATTTTAATA

* ** * *** ******* ************ * *** ************

**A)** Dendogram illustrating the similarity of the 10 gene LCAT family with EHI_065250 B) LCAT EHI_065250 sequences targeted for amplification (using the underlined primer sequences) aligned with the most similar members of the gene family. Mismatches highlighted in red. A double underline indicates the location LCAT EHI_065250 SNPs.
